# Supplementary figures and images for: Fasciola hepatica juveniles interact with the host fibrinolytic system as a potential early-stage invasion mechanism
Source: PLoS Negl Trop Dis. 2023 Apr 21;17(4):e0010936. doi: 10.1371/journal.pntd.0010936 (PMC10155961; doi:10.1371/journal.pntd.0010936)

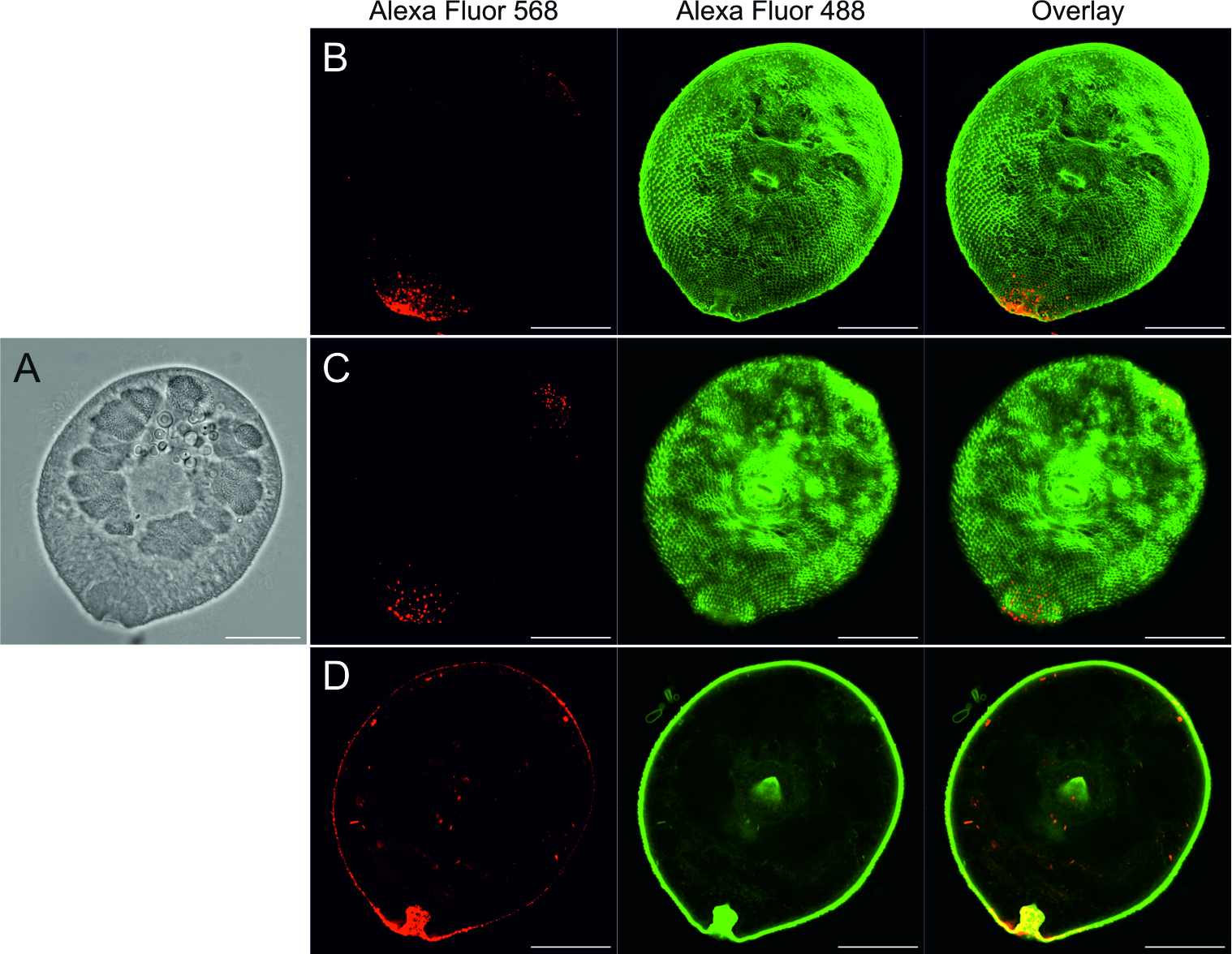

Supplement: S1 Fig — Representative image of an FhNEJ incubated in the presence of PLG (Alexa Fluor 568) and counterstained with concanavalin A (Alexa Fluor 488) to highlight the FhNEJ surface. Panels show the transmitted light image (A), the sum of all acquired Z projection slices spanning the entire FhNEJ volume (B) and the surface (C) or middle (D) plane of the projection to highlight specific PLG staining at the FhNEJ surface. Scale bars, 50 μm. (TIF) [file pntd.0010936.s002.tif]

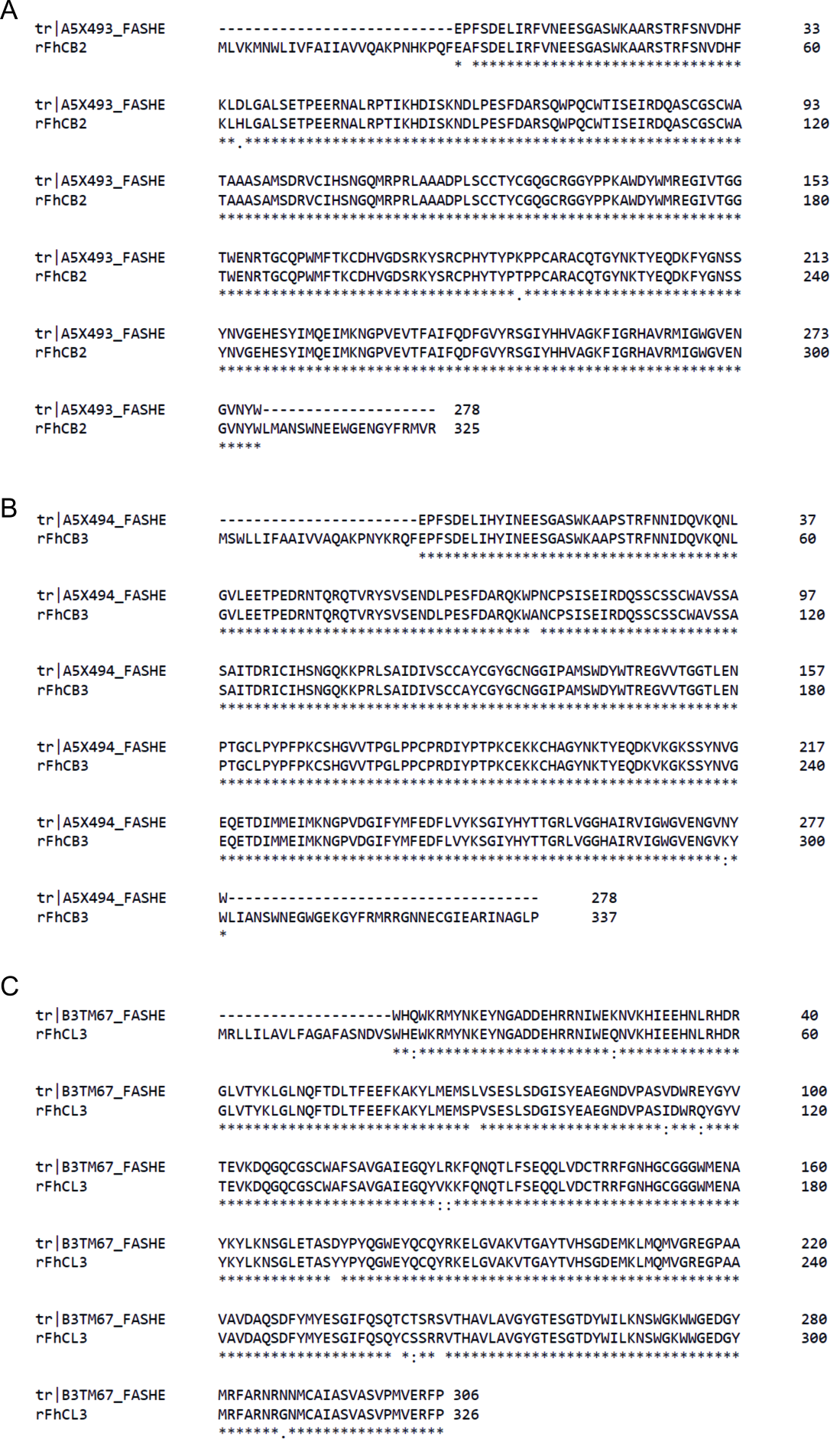

Supplement: S2 Fig — Sequence alignment between recombinant FhCB2 (A), FhCB3 (B) or FhCL3 (C) used in our validation assays (top sequences) with those identified by 2D-MS (bottom sequences) (TIF) [file pntd.0010936.s003.tif]
